# Measurement of homocysteine: a historical perspective

**DOI:** 10.3164/jcbn.19-49

**Published:** 2019-10-08

**Authors:** Sreyoshi Fatima Alam, Santosh Kumar, Paul Ganguly

**Affiliations:** 1 College of Medicine, Alfaisal University, PO Box 50927, Riyadh 11533, Kingdom of Saudi Arabia

**Keywords:** homocysteine measurement, methodology, HPLC, immunoassay, enzymatic assay

## Abstract

Elevated plasma level of homocysteine is being increasingly associated with many diseases. There is a significant interest in the development of methods to determine the total homocysteine in biologically relevant tissues. Over the years, researchers use various methods to determine the exact concentrations of homocysteine in these tissues. However, the precise method used in many studies earlier was questionable. We have reviewed various methodologies for the measurement of homocysteine. We list the commonly used methodologies currently in use to determine homocysteine levels. Through extensive literature search, we have come up with the most popular as well as the newest measurement modalities and listed them with a brief discussion of each of the methodology. In conclusion, we have presented the historical perspective of homocysteine measurement in biological fluids in this manuscript. Thus, the precise understanding of its concentration in biological fluids coupled with its importance in health and disease should justify a newer but reliable technique in the area of ongoing research in homocysteine.

## Introduction

Homocysteine, a sulfhydryl-containing amino acid, is an intermediate product in the biosynthesis of methionine and cysteine.^(1)^ Plasma homocysteine is present as 1% free thiol, 70–72% disulfide-bound to albumin and 20–30% dimer homocysteine or thiols.^(2)^ Recent years have shown a dramatic surge in research on homocysteine.^(3)^ Elevated plasma level of homocysteine is being increasingly associated with many diseases, including cardiovascular diseases,^(4–6)^ stroke,^(4,7)^ Alzheimer’s disease,^(8)^ non-alcoholic fatty liver disease (NAFLD),^(9)^ macular degeneration,^(10,11)^ renal dysfunction,^(12)^ diabetes,^(13,14)^ bone fracture^(15)^ and cancer.^(16,17)^

Some clinical studies have also shown that the increased total plasma homocysteine level correlates better than cholesterol with the risk of cardiovascular disease^(18,19)^ and proved to be a suitable tool for analysis and diagnosis of Alzheimer’s disease.^(20)^ In addition, recently the assessment of homocysteine level has been used to monitor breast cancer patients during their hormonal treatment.^(21)^ Homocysteine is also considered a biomarker of vitamin B12, B6 and folic acid status due to its important metabolic links.^(2)^ These results indicate a tremendous interest in the development of methods to determine the total homocysteine at biologically relevant concentrations.^(22)^ We still have a long way to go before it can be established as a reliable marker for health parameters, but the first step to the process involves finding suitable methodologies for the measurement of homocysteine.

## A Brief Look into the Biochemistry

Homocysteine plays a pivotal role in the methylation cycle.^(23)^ Beginning with methylation to form methionine, it progresses via *S*-adenosylation to form *S*-adenosylmethionine (SAM), which is the principal methyl donor for all methylation reactions in cells.^(23)^ The methyl group of the tertiary sulfur on SAM is transferrable and therefore capable of methylation of other substances. This methylation reaction involves energy loss, hence is irreversible. The demethylation reaction leads to the formation of *S*-adenosylhomocysteine (SAH) a thioether analogous to methionine.^(24)^ The SAM-to-SAH ratio defines the methylation potential of a cell.^(23)^ Hydrolysis of SAH leads to the formation of homocysteine and adenosine.^(24)^ This homocysteine can be used in one of two ways: in the presence of methionine deficiency, it can be re-methylated to form methionine.^(24)^ If there is no deficiency of methionine, it is instead used to produce cysteine.^(24)^

Over the years a myriad of research has delved into the involvement of homocysteine with the disease processes in the human body. This has led to a great scientific and clinical interest in the measurement of homocysteine. The recent years have seen a sharp rise in different methods of measuring plasma/serum homocysteine. Our aim in this review has been to compile and briefly compare the different methods of measurement and detection. Based on our literature search we have concluded that there have been some new methodologies introduced in this field during recent times and there seems to be lag in an updated review. There is a large variety of methods to measure and detect plasma homocysteine. Determination of homocysteine levels first began in 1962 in patients with homocystinuria.^(25)^ Since then, studies have shown that there is potential to consider homocysteine beyond the constraints of homocystinuria alone. Total homocysteine is defined as the sum of all homocysteine in plasma/serum, which includes free and protein-bound forms.^(25)^ Therefore, before proceeding to the measurement and detection, there needs to be certain steps to obtain all the homocysteine in its free form. This can be done via a variety of ways, which will be further discussed. Another point to keep under consideration is the instability of homocysteine found in whole blood as well as the postprandial and orthostatic variation in plasma homocysteine analysis.^(26)^ The acid soluble fraction of homocysteine in plasma was measured in healthy subjects with the second-generation amino acid analyzers in the mid 1970s.^(27,28)^ The first clinical studies on increased plasma homocysteine and risk of cardiovascular disease, published in the late 1970s and early 1972s, were based on this^(25)^ and since then newer methods have allowed reduction processes and measurement of total rather than only free homocysteine. For the diagnosis and monitoring of individuals with classical homocystinuria, the conventional approach has been to measure non-protein-bound homocysteine by ion exchange chromatography.^(29)^ The most important limitation of this approach is that the plasma total homocysteine concentration must exceed 60 mM (well above the normal reported range) before free homocysteine is detectable.^(30)^ A major advantage of measuring total homocysteine is that stored samples can be analyzed because total homocysteine is not altered when samples are kept frozen, even for years.^(25)^

## Various Analytical Methods: An Overview

The practicality of the existing methodologies for the determination of homocysteine levels in plasma is limited in terms of sample requirements, preparation time and instrument cost.^(22)^ Plasma levels of homocysteine can be measured by several ways. One of the newest being a new miniaturized electrochemical assay involving cytochrome c (cyt c) immobilized on gold nanoparticle (GNP) with modified screen-printed carbon electrode (SPE) used as a biosensing element.^(22)^ Another method involves enzymatic cyclic assay which measures serum homocysteine.^(31)^ These assays are based on quantitative enzymatic conversion of homocysteine to SAH. Antibodies have also been developed to recognize SAH (drawback: its indirect and cross reaction due to similarity to cysteine), allowing competitive assay of the concentration of SAH. Some simple colorimetric enzyme assays for homocysteine also allow analyses to be performed on routine clinical chemistry analyzers. These assays are based on either an enzymatic cycling assay^(32)^ or on enzymatic release of hydrogen-sulfide which reacts to form a chromogen.^(33)^

Tandem mass spectrometry and high-pressure liquid chromatography (HPLC) are other valid options for analysis of homocysteine levels.^(34)^ To summarize, the methods of quantitative determination for homocysteine include miniaturized electrochemical assay,^(22)^ amino acid analyzers, immunoassay, capillary electrophoresis, fluorescence and chromatographic techniques.^(35)^ Amino acid analyzer assays are time consuming sample analytical procedures and expensive reagents compared to chromatographic technique.^(35)^ Comparisons among these methods have shown minute bias between them, and therefore a choice between them is based on practical purposes such as cost, labor and availability.^(32–37)^ Therefore, clinical laboratories would prefer to use routine chemistry analyzers rather than immunoassay or HPLC methods. When it comes to use of appropriate detectors, both fluorescence and electrochemical detectors can be employed.^(38)^

## A Detailed Look into the Various Methods: Historical Perspective

In the early days, homocysteine was measured with amino acid analyzers using the ninhydrin reaction for detection. But these methods were insensitive and only suitable to detect homozygotes for genetic conditions leading to homocystinuria. Additionally, amino acid analyzers measure acid soluble cysteine-homocysteine mixed disulphide.^(26)^ HPLC with fluorometric detection has been the most widely used method to determine total plasma homocysteine concentrations while electrochemical detection is also often used. Electrochemical detection has the added benefit that prior derivatization of thiols is not required. As reported in the past by Ubbink,^(26)^ liquid chromatography electrospray tandem mass spectrometric assay had shown potential to become the reference method for analysis.

Other methods to measure total homocysteine include gas-liquid chromatography, capillary electrophoresis, and immunoassays. Fluorescence polarization immunoassay compares well with gas chromatography–mass spectrometry and had potential in becoming the method of choice in routine diagnostic clinical chemistry laboratories.^(26)^ Despite good selectivity and detection limits, most of them tend to require extensive sample preparation and derivatization steps prior to analysis, electrochemical, optical, chemiluminescence, fluorescence and UV-V detection which involve expensive reagents and instrumentation systems.^(22)^

The latest and most recent measurement of homocysteine involves development upon a low-cost electrochemical assay for the direct determination of homocysteine in one drop of the plasma sample using SPE modified with cyt c anchored GNP as the bio-sensing element. Modifying the electrodes with GNP, carbon nanotubes (CNT) and their combination with mediators helps improve the kinetics with improved sensitivity.^(22,39)^

## HPLC with Fluorescence Detection

A look into history shows that Refsum *et al.*^(40)^ as well as Jacobsen *et al.*^(41)^ used fully automated fluorescence assays to measure total plasma homocysteine, back in the year 1981. They used sodium borohydride (NaBH_4_) for the reduction of disulfides and protein bound homocysteine, followed by derivatization of the liberated thiols with monobromobimane. It was complicated and involved dual column HPLC system, column-switching, and two solvent delivery systems.^(41)^ Despite this, it had been a considerable step forward from the amino acid analyzers.

Araki and Sako^(42)^ separated plasma thiols within 12–20 min via reversed phase HPLC and a gradient elution program. This method was modified to allow isocratic separation within 5 min by decreasing the mobile phase buffer pH (2.1), thus ensuring that more than 50% of SBD-homocysteine carboxyl groups were protonated and this helped to increase the retention time of SBD-homocysteine and resulted in its complete resolution from SBD-cysteinylglycine. This method ensured high sample output, but the low pH of the mobile phase increased dissolution of the silica matrix.^(25)^ A silica saturation column mounted in front of the injector or a guard column may reduce this process.^(25)^

Vester and Rasmussen^(43)^ eluted the ammonium-7-fluorobenzo-2-oxa-1,3-diazole-4-sulfonic acid (SBD-F) adducts with a methanol gradient and obtained high precision of the assay by including mercaptopropionylglycine as internal standard but this significantly increased the run time. The use of internal standards should theoretically improve the recovery and precision of chromatographic methods but the extent of improvement in homocysteine analysis is uncertain^(26)^ in particular since counter studies by Accinni *et al.*^(44)^ found that the internal standards such as such as cysteamine, mercaptopropionylglycine and *N*-acetylcysteine counterintuitively showed that the precision deteriorated with internal standard use. The mechanism may relate to the different chemical behavior of the SBD-thiol adducts or to the relative increased light sensitivity of SBD-internal standard derivatives compared with the SBD-homocysteine adduct.^(26)^

Despite this the use of SBD-F as a derivatization agent has been popular, and some clinical studies have employed the assay developed by Araki and Sako^(42)^ or its modifications in the determination of plasma homocysteine concentrations. The SBD-F-based methods seem to be sensitive and specific, with no interfering reagent peaks in the chromatogram.^(25)^ It has certain drawback like low reactivity of SBD-F and the long reaction time and high temperature requirements.^(25)^

Monobromobimane (mBrB) has been used for determination of homocysteine.^(34,35)^ NaBH4 is used as a reductant. The presence of dithioerythritol during the reduction and derivatization increases the linearity of the assay in the lower range, by preventing reoxidation.^(49)^ Excess fluorescent mBrB is removed by thiol Sepharose^(25,41)^ which involves cumbersome procedures, since column switching involves 2 solvent delivery system leading to pressure surges that decreases column life.^(25)^ Refsum *et al.*^(40)^ developed a fully automated method based on NaBH4 reduction and derivatization with mBrB, to increase the reliability and it did not involve column switching and instead took advantage of the marked pH dependence of the retention of several mBrB derivatives in reversed-phase chromatography. Baseline separation of homocysteine, cysteine, and cysteinylglycine was obtained by eluting the column with an ammonium formate buffer adjusted to pH 3.65.^(45)^

Certain modifications are based on precolumn derivatization with ophthaldialdehyde followed by HPLC and fluorescence detection.^(46,47)^ Two assays have been published where plasma is treated with a reductant, homocysteine is carboxymethylated with iodoacetate before derivatization with ophthaldialdehyde, and homocysteic acid is used as internal standard. The major differences between these assays are the reductant used and the retention times (10 and 22 min). Detection of SBD-F derivative after HPLC is probably currently the most widely used assay for plasma total homocysteine.^(30)^ Dudman and colleagues^(48)^ published details of this assay using homocysteine as primary calibrator and *N*-acetylcysteine as an internal standard.

## HPLC with Post-column Derivatization

Andersson *et al.*^(49)^ developed an HPLC method for the measurement of total homocysteine using ion-pair chromatography followed by post-column derivatization and spectrophotometric detection with good precision (1.5% intra-batch; 2.5% inter-batch), and a sensitivity of less than 50 nM. The assay utilized isocratic reversed-phase ion-pair liquid chromatography at pH 2.4, post-column derivatization with 4,4'-dithiodipyridine, and colorimetric detection at 324 nm.

## HPLC with Electrochemical Detection

The electrochemical detection of homocysteine after HPLC increases sensitivity and shortens run time (approximately half that for fluorescence detection) and eliminates the need for derivatization, unfortunately this comes at a price of reduced reliability because of flow cell contamination.^(50)^ Martin *et al.*^(51)^ improved precision by using coulometric electrochemical detection with penicillamine as the internal standard.

## HPLC with Mass Spectrometry

A liquid chromatography electrospray tandem mass spectrometric (LC-MS-MS) method for the analysis of plasma and urine homocysteine concentrations has been published.^(52)^ The method uses preincubation of the sample with dithiothreitol to reduce protein-bound homocysteine, followed by LC-MS-MS analysis. It involves a short chromatography to separate homocysteine from the biological material present in the sample.^(26)^ Detection involves quadrupole mass spectrometry which indicates that the first quadrupole transmits only the parent ions of interest.^(30)^

Magera *et al.*^(52)^ reported intra-batch and inter-batch precision of 2.9 ± 5.9 and 3.6 ± 5.3% for mean total homocysteine of 3.9, 22.7 and 52.8 mM. This can potentially be used on blood spots and this makes way for use as screening purposes despite some loss of precision. The high cost of the instrument can be weighed in with fast sample turnover.^(30)^ Tuschl *et al.*^(34)^ modified the tandem MS method by using less sample volume and shorter dithiothreitol (DTT) reduction time; however, the recovery at 8.9 µM was only 49% and the limit of quantitation (LOQ) was about 4 µM.

Jiang *et al.*^(35)^ developed and validated a fast and sensitive homocysteine and methionine assay using LC tandem mass spectrometry for high volume routine lab analysis, using a small amount plasma sample (50 µl), short preparation time (<1 h, no derivatization) and short running time (4 min). The simultaneous measurement of methionine and total homocysteine improves the diagnosis of disorders involving Sulphur amino acids. This also has wider analytical measurement ranges.^(35)^

A paper published by Espina *et al.*^(53)^ proposed the use of ebselen, a selenium containing labelling agent for derivatization of reactive sulfhydryl group of the homocysteine molecule in its reduced form. The selenium atom in the molecule allows the use of inductively coupled plasma mass spectrometry (ICP-MS) as a sensitive and selective selenium detector. HPLC coupled with ICP-MS can determine Se-derivatized reduced homocysteine (detection limit of 9.6 nM) in real samples.

## Enzymatic and Immunoassays

The earliest forms of current immunoassays for the measurement of plasma homocysteine concentrations involved condensation of homocysteine with ^14^C-adenosine catalyzed by *S*-adenosyl-homocysteine hydrolase. The labeled SAH is separated from ^14^C-adenosine and its radioactive response, which is directly proportional to the homocysteine concentration in plasma.^(54)^ Ueland *et al.*^(55)^ described this as a radioenzymatic assay. It used dithioerythritol as reductant, and radioactive SAH was quantified by HPLC and scintillation counting.^(55)^ This assay can also be carried out with unlabeled adenosine and ultraviolet detection, thin-layer chromatography or paper chromatography instead of HPLC.^(54,55)^ Thin-layer chromatography or paper chromatography require inexpensive equipment and can be established with ease in most laboratories. The drawbacks involve lengthy enzyme incubation, protein precipitation, neutralization, and centrifugation. Another shortcoming is the limited range of this assay, due to the consumption of radioactive adenosine.^(25)^ This method showed improved sensitivity relative to the conventional ion-exchange chromatography using an amino acid analyzer but has been replaced by immunoassays that do not require radiolabeled substrates.^(30)^

Since the discovery of direct HPLC assays this method has become highly obsolete.^(26)^ But this method has paved the pathway for the enzymatic conversion of homocysteine to SAH after mouse monoclonal antibodies were developed against SAH.^(26)^ This allowed the development of the infrastructure of immunoassays in which the amount of SAH was measured in a competitive enzyme immunoassay and its precision is comparable to that of HPLC.^(26)^ Building on this previously established infrastructure, a fluorescence polarization immunoassay has been developed^(26)^ where homocysteine is selectively converted to SAH, and competition between SAH is established and a fluoresceinated SAH analogue for binding to a monoclonal antibody forms the basis of an automated assay method that can be performed on an IMX analyzer. IMX analyzer and microplate readers are common in most laboratories. Therefore, randomized controlled clinical trials if performed and if they show clinical benefit of homocysteine-lowering therapy this might reflect as an increased demand for these assays.^(26)^

## Enzyme-linked Microplate Immunoassay

To build upon the topic discussed, enzyme-linked immunoassay (EIA) in microtitre format has been of great importance in this field. Frantzen *et al.*^(56)^ successfully reduced homocysteine [with l-dithiothreitol (DTT)] and synthesized SAH hydrolase and adenosine in a single incubation step. This was followed by inhibition of SAH hydrolase and removal of excess adenosine. The designed immunoassay is carried out in microtiter plates pre-coated with a bovine serum albumin-SAH (BSA-SAH) conjugate, with added SAH hydrolase-treated samples and monoclonal mouse anti-SAH antibody. Horseradish peroxidase-conjugated antibody is used for competitive binding, with tetra-methyl benzidine as the substrate. The horseradish peroxidase-conjugated reaction is quantified spectrophotometrically at 450 nm. Cystathionine, methionine, adenosine or cysteine showed no interference in the assay but 5-adenosyl methionine at less than 10 mM gave falsely elevated homocysteine through cross-reactivity with the anti-SAH antibody, but as this exceeds reported concentrations of plasma SAH it is not expected to be of significance.^(30)^ The study also reported within-run and between run precision of less than 5.5% and less than 8.2%, respectively, over the homocysteine range 8 to 27 mM. The method has a practical advantage over other methods since it does not involve cumbersome, expensive and sophisticated equipment, thus making it more feasible for laboratories.

## Fluorescence Polarization Immunoassay

The enzyme-linked immunoassay has been adapted for full automation on the Abbott IMx analyzer (Abbott, North Chicago, IL) where the microtiter plate method has been replaced by fluorescence polarization for the detection of antibody-bound SAH.^(30)^ The reduction of homocysteine and conversion to SAH is similar to EIA. Shipchandler and Moore^(57)^ reported full automation of the immunoassay for plasma total homocysteine within a range from 1.8 to 8.0% and 1.8 to 6.4% for intra- and inter-assay, respectively, over the concentration range 3.67 ± 60 mM (derived from the measurement of calibrators not plasma samples). Precision across three instruments ranged from 5.8 to 10.2% over the concentration range 5 ± 33.2 mM. Recovery of l-homocysteine added to plasma as l-homocysteine averaged 97.1 and 99.9% for two plasma samples for homocysteine concentrations up to 29 mM. The same assay was evaluated by Yu *et al.*^(58)^ and between-run CV were reduced to an impressive 2.9, 0.8 and 1.7% at total homocysteine 7.0, 12.5 and 25 mM, respectively. This analyzer can process at least 20 samples per hour, which is comparable to the EIA assay but superior to manual HPLC.^(30)^

Tewari *et al.*^(59)^ used Centaur homocysteine assay which involves three-step procedure which begins with reduction of homocysteine disulfides followed by enzymatic conversion to SAH and finally quantitation of SAH in a competitive immunoassay (labeled anti-SAH antibody: magnetic particles coupled with SAH).

Homocysteine competes with biotinylated SAH coupled to avidin coated paramagnetic particles (PMP), for a limited amount of anti-SAH monoclonal antibody labeled with acridinium ester, used as a chemiluminescent tracer. This immunoreaction is followed by magnetic separation and washing of PMP with deionized water to remove chemiluminescent tracer not bound to the solid phase. Alkaline hydrogen peroxide is added to initiate the chemiluminescent reaction and then the chemiluminescence is measured by a luminometer as relative light units (RLU). These RLUs are compared with a calibration curve to compute homocysteine concentration in the patient’s sample. Some studies have shown that homocysteine measured directly via immunoassay techniques can lead to antibodies raised against homocysteine to cross react with cysteine, particularly due to its high levels in plasma. However, monoclonal antibodies raised against SAH are not cross-reactive with cysteine.^(59)^

Compared to fluorescent polarization immunoassay HPLC this study produced linear regression equations with slopes between 0.95 and 1.0. The study concluded that the Centaur homocysteine assay is a sensitive and precise assay for the measurement of homocysteine.^(59)^

## N Latex Homocysteine Immunonephelometric Method

Zappacosta *et al.*^(60)^ evaluated an immunonephelometric method for homocysteine determination. As usual the first step is disulfide reduction to free homocysteine by dithiothreitol and enzymatic conversion to SAH. Unlike the previously discussed methodologies, SAH competes in the reaction with thyroglobulin (TG) conjugated *S*-adenosyl-cysteine (SAC) for bonding to anti-SAH antibodies bound to polystyrene particles. In the presence of SAH, a weaker aggregation of particles occurs, whereas in its absence a complete aggregation of the polystyrene particles is induced by the TG-conjugated SAC. The SAH content in the reaction mixture is inversely related to the scattered light signal. The result is evaluated by comparison with a known concentration of a standard.

The method was compared with a HPLC reference method and an automated immunoassay method. Recovery range was 96.4–104.2%, limit of detection was 0.5 µM and total imprecision ranged from 5.0 to 7.6%. For the comparison study, the immunonephelometric method showed a good correlation both with HPLC (Y = 1.02X – 0.71, R^2^ = 0.99) and with immunoassay method (Y = 1.003X + 0.06, R^2^ = 0.98).

The nephelometers is abundantly available in most clinical laboratories, therefore, based on the analytical reliability of the immunonephelometric method, relative availability of instrumentation, Zappacosta *et al.*^(60)^ proposed this method as a concrete alternative to the commercially available methods for homocysteine measurement.

Another method evaluated by Roberts and Roberts^(32)^ involved a recombinant enzymatic cycling assay for total homocysteine on an automated chemistry analyzer. Homocysteine is converted to l-cystathionine by cystathionine β-synthase (CBS). l-Cystathionine is converted back to homocysteine and pyruvate by cystathionine β lyase (CBL). Lactate dehydrogenase (LD) catalyzes the reduction of pyruvate and the concurrent oxidation of NADH to NAD^+^. The resulting change in absorbance is measured at 340 nm. The concentration of homocysteine in the sample is considered directly proportional to the amount of NADH oxidized to NAD^+^.^(32)^ These results concluded that this assay for total homocysteine analysis performs well for risk stratification for vascular diseases and assessment of the homocysteine metabolic pathway.

Fu *et al.*^(61)^ designed two assays. An enzymatic assay that determines levels of homocysteine in multiple samples within 30 min at levels from 5 to 50 pmol using a spectrophotometer and a second 5-fold more sensitive assay that follows the enzymatic catalyzed solvent exchange of protons on glycine, which requires a scintillation counter. Both the spectrophotometric and the radiometric methods are based on methionine synthase conversion of 5-methyltetrahydrofolate to tetrahydrofolate. In the spectrophotometric method the tetrahydrofolate is used at catalytic levels by three enzymes to ultimately generate NADPH from NADP. In the radiometric assay the rate of proton exchange can be related to the level of homocysteine in a biological sample.^(61)^

Tan *et al.*^(33)^ reported an enzymatic assay on the Hitachi 912 automatic chemistry analyzer. The principle of the assay is that rHCYase produces H_2_S from Homocysteine and that the H_2_S is quantified by its reaction with *N*,*N*-dibutyl-phenylenediamine, which produces a chromophore. The assay is linear to at least 72 µM homocysteine, as determined visually after measurement of various amounts of homocysteine in phosphate-buffered saline. The within-run imprecision (CV) was 4.8%, 3.0%, and 4.5% at 8.9 µM, 14.9 µM, and 25 µM homocysteine (*n* = 8), respectively.^(33)^ The between-assay CV over 10 days was 7.8%, 5.9%, and 4.9% at 8.8, 15, and 25 µM, respectively.^(33)^ These imprecisions are within ranges reported for currently used assays, including the Bio-Rad HPLC assay. 121 plasma samples were assessed with the enzymatic assay on the Hitachi 912 (y) and with the Bio-Rad HPLC and the differences were not significantly correlated with homocysteine concentration.^(33)^

Nexo *et al.*^(36)^ looked at novel homocysteine assays for the European Demonstration Project involving six centers in four countries. Two immunological methods: fluorescence polarization immunoassay (FPIA) and the EIA, were compared with HPLC and gas chromatography–mass spectrometry (GC-MS). They looked at linearity and precision in five plasma samples, established a correlation using patients’ samples and assessed long-term performance. Results obtained by GC-MS were set as the standard for comparison. In general, all methods showed linearity throughout the 5–45 µM range. The FPIA method showed low imprecision throughout the range. Thus, based on the accuracy of the FPIA method, it is feasible to directly compare values obtained in different laboratories. This is significant for both clinical studies and routine clinical chemistry. The EIA has a high throughput and low sample volume requirement, but higher imprecision than the FPIA, more if the manual EIA is employed. Therefore, the EIA is more suited for screening purposes. The results of immunological methods compared well with results obtained with the comparison method, and no systematic bias of significant magnitude was noted.^(36)^

## Electrochemical Assay with Screen-printed Electrodes with Cyt c Anchored Gold Nanoparticles

Last but not the least, is a summarized discussion on the latest methodology, using electrochemical assay. The previously established methods are limited by sample requirements, preparation time and instrument cost.^(22)^ Madasamy *et al.*^(22)^ developed a low-cost electrochemical assay for the direct determination of homocysteine in one drop of the plasma sample using SPE modified with cyt c anchored GNP as the biosensing element, mainly based on the reduction of cyt c with thiols.

Quantification is based on the electrochemical oxidation of homocysteine by the Fe^3+^/Fe^2+^ crevice of cyt c, seen at a potential of +0.56 V. For human plasma, the quantification required reduction with NaBH_4_. The practical application of the present assay is validated via measurement of homocysteine in blood plasma samples, in which the results were comparable to standard methods.^(22)^ The selectivity is improved by eliminating the common interfering biological substrates using a Nafion membrane.^(22)^ This biosensor has good repeatability, reproducibility (2.77% SD) and high stability (75% of its initial current response after 4 weeks).^(22)^

Separation based techniques such as HPLC, GC-MS and capillary electrophoresis require extensive sample preparation and derivatization prior to sample analysis. Methods like chemiluminescence, fluorescence, and UV/Vis detection protocols require expensive reagents and systems.^(22)^ Electrochemical biosensor techniques have recently been considered as an alternative for the measurement. Modifying the electrodes with nanomaterials, such as GNP, CNT and their combination with mediators to improve the kinetics leads to improved sensitivity.^(22)^

Enzymatic biosensors employ quite a unique technique of direct electron transfer (DET) to or from a deep active site of enzyme to the conducting electrode surface.^(22)^ The GNP modified surface provides the infrastructure for binding of biomolecules, without losing its biological activity.^(22)^ It also reduces the electron transfer distance between the active site of cytochrome c and the electrode surface leading to a nA range sensitivity.^(22)^ The accuracy of the present assay was tested by determining the recovery of known amounts of homocysteine added to the real samples.^(22)^ The good recovery values from 92.6 to 102.9% indicated a good accuracy.^(22)^

The dynamic linear range of the biosensing element suggests its applicability for the hyperhomocysteinemia ranges of moderate (16–30 µM), medium (30–100 µM) and severe (>100 µM) and this indicates that the electrochemical assay has a promising potential for homocysteine measurements.^(22)^

## Conclusion

Homocysteine has been gaining importance with a satisfactory momentum through the passage of time. The possibility of its importance in routine chemistry is well within view of the horizon. Hence, along with the established measurement methods, there is a great need for new and commercially feasible methods for its measurement. The scientific world shows good promise in this regard. However, in spite of the fact that this review has brought some insight into the various methods that are available for the measurement of homocysteine, HPLC is still a common methodology practiced at the present time.^(62)^ The authors highly encourage readers to delve into each of the methodologies for a deeper understanding of each.

## Author Contributions

SFA: manuscript writing and literature review, PG: manuscript writing and literature review, SK: literature review.

## Abbreviations

BSA-SAH: bovine serum albumin-*S*-adenosylhomocysteine
CBL: cystathionine β lyase
CNT: carbon nanotubes
cyt c: cytochrome c
DET: direct electron transfer
DTT: dithiothreitol
EIA: enzyme-linked immunoassay
FPIA: fluorescence polarization immunoassay
GC-MS: gas chromatography–mass spectrometry
GNP: gold nanoparticle
HPLC: high pressure/performance liquid chromatography
ICP-MS: inductively coupled plasma mass spectrometry
LC-MS-MS: liquid chromatography electrospray tandem mass spectrometric
LD: lactate dehydrogenase
LOQ: limit of quantitation
mBrB: monobromobimane
NaBH4: sodium borohydride
NAD: nicotinamide adenine dinucleotide
NADP: nicotinamide adenine dinucleotide phosphate
PMP: paramagnetic particles
RLU: relative light units
SAH: *S*-adenosylhomocysteine
SAM: *S*-adenosylmethionine
SBD-F: ammonium-7-fluorobenzo-2-oxa-1,3-diazole-4-sulfonic acid
SPE: screen-printed carbon electrode

## References

[1] Nobel Media AB The Nobel Prize in Chemistry 1955 2013. http://www.nobelprize.org/nobel_prizes/chemistry/laureates/1955/

[2] Faeh D, Chiolero A, Paccaud F Homocysteine as a risk factor for cardiovascular disease: should we (still) worry about? Swiss Med Wkly 2006; 136: 745–756. 17225194 10.4414/smw.2006.11283

[3] Venes D Taber’s Cyclopedic Medical Dictionary (21st ed.). Philadelphia: F. A. Davis, 2005; 1089.

[4] Ganguly P, Alam SF Role of homocysteine in the development of cardiovascular disease. Nutr J 2015; 14: 6. 25577237 10.1186/1475-2891-14-6 PMC4326479

[5] Takemoto Y Central cardiovascular actions of L-homocysteine microinjected into ventrolateral medullary autonomic areas of the rat. Amino Acids 2016; 48: 2215–2225. 27178024 10.1007/s00726-016-2259-3

[6] Balogh E, Maros T, Daragó A, et al Plasma homocysteine levels are related to medium-term venous graft degeneration in coronary artery bypass graft patients. Anatol J Cardiol 2016; 16: 868–873. 27147400 10.14744/AnatolJCardiol.2016.6738 PMC5324890

[7] Shi Z, Guan Y, Huo YR, et al Elevated total homocysteine levels in acute ischemic stroke are associated with long-term mortality. Stroke 2015; 46: 2419–2425. 26199315 10.1161/STROKEAHA.115.009136 PMC4542568

[8] Morris MS Homocysteine and Alzheimer’s disease. Lancet Neurol 2003; 2: 425–428. 12849121 10.1016/s1474-4422(03)00438-1

[9] Dai H, Wang W, Tang X, et al Association between homocysteine and non-alcoholic fatty liver disease in Chinese adults: a cross-sectional study. Nutr J 2016; 15: 102. 27955646 10.1186/s12937-016-0221-6 PMC5153832

[10] Ghosh S, Saha M, Das D A study on plasma homocysteine level in age-related macular degeneration. Nepal J Ophthalmol 2013; 5: 195–200. 24172554 10.3126/nepjoph.v5i2.8728

[11] Huang P, Wang F, Sah BK, et al Homocysteine and the risk of age-related macular degeneration: a systematic review and meta-analysis. Sci Rep 2015; 5: 10585. 26194346 10.1038/srep10585 PMC4508850

[12] Levi A, Cohen E, Levi M, Goldberg E, Garty M, Krause I Elevated serum homocysteine is a predictor of accelerated decline in renal function and chronic kidney disease: a historical prospective study. Eur J Intern Med 2014; 25: 951–955. 25457436 10.1016/j.ejim.2014.10.014

[13] Gong T, Wang J, Yang M, et al Serum homocysteine level and gestational diabetes mellitus: a meta-analysis. J Diabetes Investig 2016; 7: 622–628. 10.1111/jdi.12460 PMC4931215 27180921

[14] Alis R, Sanchis-Gomar F, Pareja-Galeano H, et al Association between irisin and homocysteine in euglycemic and diabetic subjects. Clin Biochem 2014; 47: 333–335. 25192692 10.1016/j.clinbiochem.2014.08.017

[15] Enneman AW, Swart KM, Zillikens MC, et al The association between plasma homocysteine levels and bone quality and bone mineral density parameters in older persons. Bone 2014; 63: 141–146. 24631997 10.1016/j.bone.2014.03.002

[16] Xu W, Cheng Y, Zhu H Evaluation of an association of blood homocysteine levels with gastric cancer risk from 27 case-control studies. Medicine (Baltimore) 2016; 95: e3700. 27196483 10.1097/MD.0000000000003700 PMC4902425

[17] Tastekin D, Erturk K, Bozbey HU, et al Plasma homocysteine, folate and vitamin B12 levels in patients with lung cancer. Exp Oncol 2015; 37: 218–222. 26422108

[18] Ozkan Y, Ozkan E, Simşek B Plasma total homocysteine and cysteine levels as cardiovascular risk factors in coronary heart disease. Int J Cardiol 2002; 82: 269–277. 11911915 10.1016/s0167-5273(02)00010-4

[19] Wierzbicki AS Homocysteine and cardiovascular disease: a review of the evidence. Diab Vasc Dis Res 2007; 4: 143–150. 17654449 10.3132/dvdr.2007.033

[20] Kim H, Lee KJ Serum homocysteine levels are correlated with behavioral and psychological symptoms of Alzheimer’s disease. Neuropsychiatr Dis Treat 2014; 10: 1887–1896. 25336954 10.2147/NDT.S68980 PMC4200070

[21] Lin J, Lee IM, Song Y, et al Plasma homocysteine and cysteine and risk of breast cancer in women. Cancer Res 2010; 70: 2397–2405. 20197471 10.1158/0008-5472.CAN-09-3648 PMC2840179

[22] Madasamy T, Santschi C, Martin OJ A miniaturized electrochemical assay for homocysteine using screen-printed electrodes with cytochrome c anchored gold nanoparticles. Analyst 2015; 140: 6071–6078. 26198379 10.1039/c5an00752f

[23] Hankey GJ, Eikelboom JW Homocysteine and vascular disease. Lancet 1999; 354: 407–413. 10437885 10.1016/S0140-6736(98)11058-9

[24] Handy DE, Castro R, Loscalzo J Epigenetic modifications: basic mechanisms and role in cardiovascular disease. Circulation 2011; 123: 2145–2156. 21576679 10.1161/CIRCULATIONAHA.110.956839 PMC3107542

[25] Ueland PM, Refsum H, Stabler SP, Malinow MR, Andersson A, Allen RH Total homocysteine in plasma or serum: methods and clinical applications. Clin Chem 1993; 39: 1764–1779. 8375046

[26] Ubbink JB Assay methods for the measurement of total homocyst(e)ine in plasma. Semin Thromb Hemost 2000; 26: 233–241. 11011841 10.1055/s-2000-8468

[27] Gupta VJ, Wilcken DE The detection of cysteine-homocysteine mixed disulphide in plasma of normal fasting man. Eur J Clin Invest 1978; 8: 205–207. 100323 10.1111/j.1365-2362.1978.tb00853.x

[28] Wilcken DE, Gupta VJ Cysteine-homocysteine mixed disulphide: differing plasma concentrations in normal men and women. Clin Sci (Lond) 1979; 57: 211–215. 477256 10.1042/cs0570211

[29] Walker V, Mills GA Quantitative methods for amino acid analysis in biological fluids. Ann Clin Biochem 1995; 32 (Pt 1): 28–57. 7762950 10.1177/000456329503200103

[30] Powers HJ, Moat SJ Developments in the measurement of plasma total homocysteine. Curr Opin Clin Nutr Metab Care 2000; 3: 391–397. 11151085 10.1097/00075197-200009000-00011

[31] Zeng Y, Li M, Chen Y, Wang S Homocysteine, endothelin-1 and nitric oxide in patients with hypertensive disorders complicating pregnancy. Int J Clin Exp Pathol 2015; 8: 15275–15279. 26823880 PMC4713666

[32] Roberts RF, Roberts WL Performance characteristics of a recombinant enzymatic cycling assay for quantification of total homocysteine in serum or plasma. Clin Chim Acta 2004; 344: 95–99. 15149876 10.1016/j.cccn.2004.02.013

[33] Tan Y, Sun X, Tang L, et al Automated enzymatic assay for homocysteine. Clin Chem 2003; 49 (6 Pt 1): 1029–1030. 12766025 10.1373/49.6.1029

[34] Tuschl K, Bodamer OA, Erwa W, Mühl A Rapid analysis of total plasma homocysteine by tandem mass spectrometry. Clin Chim Acta 2005; 351: 139–141. 15563882 10.1016/j.cccn.2004.08.016

[35] Jiang Y, Mistretta B, Elsea S, Sun Q Simultaneous determination of plasma total homocysteine and methionine by liquid chromatography-tandem mass spectrometry. Clin Chim Acta 2017; 464: 93–97. 27845054 10.1016/j.cca.2016.11.017

[36] Nexo E, Engbaek F, Ueland PM, et al Evaluation of novel assays in clinical chemistry: quantification of plasma total homocysteine. Clin Chem 2000; 46 (8 Pt 1): 1150–1156. 10926896

[37] Dou C, Xia D, Zhang L, et al Development of a novel enzymatic cycling assay for total homocysteine. Clin Chem 2005; 51: 1987–1989. 16299897 10.1373/clinchem.2005.053421

[38] Albu C, Litescu SC, Radu GL, Aboul-Enein HY Validated HPLC-Fl method for the analysis of S-adenosylmethionine and S-adenosylhomocysteine biomarkers in human blood. J Fluoresc 2013; 23: 381–386. 23408091 10.1007/s10895-013-1173-2

[39] Gholami-Orimi F, Taleshi F, Biparva P, et al Voltammetric determination of homocysteine using multiwall carbon nanotube paste electrode in the presence of chlorpromazine as a mediator. J Anal Methods Chem 2012; 2012: 902184. 22675657 10.1155/2012/902184 PMC3364601

[40] Refsum H, Ueland PM, Svardal AM Fully automated fluorescence assay for determining total homocysteine in plasma. Clin Chem 1989; 35: 1921–1927. 2776317

[41] Jacobsen DW, Gatautis VJ, Green R Determination of plasma homocysteine by high-performance liquid chromatography with fluorescence detection. Anal Biochem 1989; 178: 208–214. 2729575 10.1016/0003-2697(89)90381-3

[42] Araki A, Sako Y Determination of free and total homocysteine in human plasma by high-performance liquid chromatography with fluorescence detection. J Chromatogr 1987; 422: 43–52. 3437026 10.1016/0378-4347(87)80438-3

[43] Vester B, Rasmussen K High performance liquid chromatography method for rapid and accurate determination of homocysteine in plasma and serum. Eur J Clin Chem Clin Biochem 1991; 29: 549–554. 1760484 10.1515/cclm.1991.29.9.549

[44] Accinni R, Campolo J, Bartesaghi S, et al High-performance liquid chromatographic determination of total plasma homocysteine with or without internal standards. J Chromatogr A 1998; 828: 397–400. 9916320 10.1016/s0021-9673(98)00661-x

[45] Fiskerstrand T, Refsum H, Kvalheim G, Ueland PM Homocysteine and other thiols in plasma and urine: automated determination and sample stability. Clin Chem 1993; 39: 263–271. 8432015

[46] Fermo I, Arcelloni C, De Vecchi E, Viganó S, Paroni R High-performance liquid chromatographic method with fluorescence detection for the determination of total homocyst(e)ine in plasma. J Chromatogr 1992; 593: 171–176. 1639902 10.1016/0021-9673(92)80283-z

[47] Hyland K, Bottiglieri T Measurement of total plasma and cerebrospinal fluid homocysteine by fluorescence following high-performance liquid chromatography and precolumn derivatization with o-phthaldialdehyde. J Chromatogr 1992; 579: 55–62. 1447351 10.1016/0378-4347(92)80362-t

[48] Dudman NP, Guo XW, Crooks R, Xie L, Silberberg JS Assay of plasma homocysteine: light sensitivity of the fluorescent 7-benzo-2-oxa-1, 3-diazole-4-sulfonic acid derivative, and use of appropriate calibrators. Clin Chem 1996; 42: 2028–2032. 8969644

[49] Andersson A, Isaksson A, Brattström L, Hultberg B Homocysteine and other thiols determined in plasma by HPLC and thiol-specific postcolumn derivatization. Clin Chem 1993; 39: 1590–1597. 8353942

[50] Malinow MR, Kang SS, Taylor LM, et al Prevalence of hyperhomocyst(e)inemia in patients with peripheral arterial occlusive disease. Circulation 1989; 79: 1180–1188. 2785871 10.1161/01.cir.79.6.1180

[51] Martin SC, Tsakas-Ampatzis I, Bartlett WA, Jones AF Measurement of plasma total homocysteine by HPLC with coulometric detection. Clin Chem 1999; 45: 150–152. 9895359

[52] Magera MJ, Lacey JM, Casetta B, Rinaldo P Method for the determination of total homocysteine in plasma and urine by stable isotope dilution and electrospray tandem mass spectrometry. Clin Chem 1999; 45: 1517–1522. 10471655

[53] Espina JG, Montes-Bayón M, Blanco-González E, Sanz-Medel A Determination of reduced homocysteine in human serum by elemental labelling and liquid chromatography with ICP-MS and ESI-MS detection. Anal Bioanal Chem 2015; 407: 7899–7906. 26362154 10.1007/s00216-015-8956-z

[54] Refsum H, Helland S, Ueland PM Radioenzymic determination of homocysteine in plasma and urine. Clin Chem 1985; 31: 624–628. 3978799

[55] Ueland PM, Helland S, Broch OJ, Schanche JS Homocysteine in tissues of the mouse and rat. J Biol Chem 1984; 259: 2360–2364. 6698970

[56] Frantzen F, Faaren AL, Alfheim I, Nordhei AK Enzyme conversion immunoassay for determining total homocysteine in plasma or serum. Clin Chem 1998; 44: 311–316. 9474030

[57] Shipchandler MT, Moore EG Rapid fully automated measurement of plasma homocyst(e)ine with the Abbott IMx analyzer. Clin Chem 1995; 41: 991–994. 7600701

[58] Yu HH, Joubran R, Asmi M, et al Agreement among four homocysteine assays and results in patients with coronary atherosclerosis and controls. Clin Chem 2000; 46: 258–264. 10657383

[59] Tewari PC, Zhang B, Bluestein BI Analytical and clinical evaluation of the Bayer ADVIA Centaur homocysteine assay. Clin Chim Acta 2004; 342: 171–178. 15026278 10.1016/j.cccn.2003.12.025

[60] Zappacosta B, Persichilli S, Minucci A, et al Evaluation of a new enzymatic method for homocysteine measurement. Clin Biochem 2006; 39: 62–66. 16297375 10.1016/j.clinbiochem.2005.10.001

[61] Fu TF, di Salvo M, Schirch V Enzymatic determination of homocysteine in cell extracts. Anal Biochem 2001; 290: 359–365. 11237340 10.1006/abio.2001.4994

[62] Maruyama K, Eshak ES, Kinuta M, et al Association between vitamin B group supplementation with changes in % flow-mediated dilatation and plasma homocysteine levels: a randomized controlled trial. J Clin Biochem Nutr 2019; 64: 243–249. 31138959 10.3164/jcbn.17-56 PMC6529698

